# Patchy Earliest Activation on Near‐Field Annotation Mapping: A Pitfall in Ablation of Premature Ventricular Contractions Originating From the Right Ventricular Outflow Tract

**DOI:** 10.1002/joa3.70350

**Published:** 2026-04-20

**Authors:** Yusuke Sakamoto, Hiroyuki Osanai, Eiji Yoshida

**Affiliations:** ^1^ Department of Cardiology Tosei General Hospital Seto Aichi Japan

**Keywords:** catheter ablation, first deflection mapping, near‐field mapping, premature ventricular complexes, right ventricular outflow tract

## Abstract

Concordance between FD and NF mapping is associated with successful RVOT ablation of PVCs. Discordant or patchy NF activation patterns suggest distant origins and may be linked to ablation failure. Integrating NF activation patterns provides a more comprehensive interpretation beyond ΔFN.

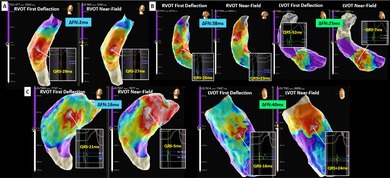

A subset of premature ventricular complexes (PVCs) presumed to originate from the right ventricular outflow tract (RVOT) actually arises from the left ventricular outflow tract (LVOT), contributing to ablation failure [1]. Recently, mapping strategies incorporating Near‐Field (NF) annotation algorithms alongside traditional First Deflection (FD) annotation have been reported. The temporal disparity between FD and NF annotations, along with the spatial relationship of their earliest activation sites, as identified on a three‐dimensional mapping system, can provide valuable insight into the PVC origin and may predict ablation success [2, 3].

We compared 20 patients who achieved successful RVOT PVC ablation with four patients in whom RVOT ablation failed but was followed by successful LVOT ablation. PVC mapping was performed using the Advisor HD Grid Multipolar Mapping Catheter with the EnSite X Mapping System (electrogram filter: 30–300 Hz). Annotation points were automatically assigned to the FD and NF signals by adjusting the system annotation settings. Electrogram analysis and measurement of the spatial distance between the earliest activation sites were conducted on the three‐dimensional mapping system. Ablation success was defined as the complete elimination of PVCs, whereas failure was defined as the inability to suppress PVCs. The number of applications and the decision to transition to LVOT mapping or ablation were left to the operator's discretion, based on the clinical context. The temporal difference between FD and NF annotations (ΔFN) was significantly smaller in successful cases (10.1 ± 7.8 ms vs. 28.0 ± 10.3 ms, *p* = 0.021). Similarly, the mean spatial distance between the earliest activation sites identified by FD and NF mapping was shorter in successful cases (4.2 ± 5.2 mm vs. 10.0 ± 6.5 mm, *p* = 0.03). These findings indicated that greater concordance between FD and NF mapping is associated with successful RVOT ablation. In cases of failed RVOT ablation, two patients exhibited a clearly defined centrifugal activation pattern on FD annotation, while NF annotation demonstrated a patchy distribution. The results of the two algorithms were discordant. In this study, a patchy NF activation pattern was defined as a distribution in which a single clear centrifugal activation pattern could not be identified and multiple earliest activation sites were observed. Figure 1A shows a typical successful RVOT ablation case. ΔFN was 2 ms, and the earliest activation sites identified by both algorithms were concordant. Ablation at this site successfully eliminated the PVC. As observed in this case, successful RVOT ablation was associated with a small ΔFN, suggesting that the PVC origin was located close to the endocardial surface. Accordingly, both mapping algorithms demonstrated a centrifugal activation pattern, and the earliest activation sites were spatially concordant.

**FIGURE 1:**
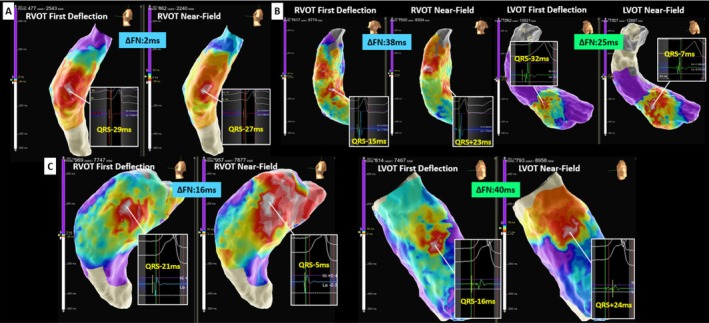
Relationship between first deflection (FD) and near‐field (NF) annotation mapping in premature ventricular complex ablation. (A) Successful right ventricular outflow tract (RVOT) ablation case. The earliest activation sites of FD and NF were concordant. (B) Failed RVOT ablation case. The earliest activation sites of FD and NF were not precisely localized. Ablation at the left ventricular outflow tract (LVOT) was successful. (C) Discordant mapping case. FD identified an apparent earliest site, whereas NF mapping exhibited a patchy distribution. Ablation at the LVOT was successful. This discordance highlights the potential limitation of relying solely on FD annotation and underscores the importance of integrating NF activation patterns for accurate localization and prediction of ablation success.

Figure 1B illustrates a failed RVOT ablation case in which both mapping algorithms suggested a remote site origin and were unable to precisely localize the earliest activation site. The ΔFN was 38 ms, and the spatial distance between the earliest sites was 19 mm. Subsequent ablation at the left coronary cusp (LCC)–right coronary cusp junction was successful, with a reduced ΔFN of 25 ms and concordant earliest activation sites. Consistent with this observation, a patchy activation pattern on both mapping algorithms indicated that the PVC origin was distant from the mapped region, and RVOT ablation was unsuccessful in all such cases.

Figure 1C displays a case with discordant algorithm results. FD annotation identified a clear apparent origin, whereas NF annotation demonstrated a patchy distribution. ΔFN was 16 ms, and the spatial distance between the earliest sites was 4 mm. Ablation at the FD‐defined site was ineffective; however, ablation at the LCC was successful, with ΔFN of 40 ms and concordant earliest activation sites.

These observations may suggest that ΔFN was influenced not only by the spatial distance from the PVC origin but also by the conduction time from the origin to the endocardial surface. Activation may spread before reaching the endocardial surface, particularly when the PVC origin is located intramurally or on the LVOT side, resulting in a broader activation area that includes bystander regions. This may produce a patchy NF activation pattern and contribute to discordance between FD and NF mapping. Even in cases with longer ΔFN, concordance between FD and NF mapping may indicate proximity to the origin and an increased likelihood of successful ablation. Conversely, a relatively short ΔFN combined with a patchy NF pattern may be consistent with an origin at an intermediate distance, where the earliest FD activation likely represents far‐field signals and multiple patchy bystander sites appear on NF mapping, suggesting a high likelihood of RVOT ablation failure. The main message of this study, while building on previous reports, is that ΔFN alone may not be sufficient to determine the true origin of PVCs or predict ablation success. In particular, the presence of cases in which RVOT ablation fails despite relatively small ΔFN values suggests the importance of integrating NF mapping activation patterns for a more comprehensive interpretation. However, these findings should be interpreted with caution. Given the limited sample size, particularly in the subgroup of failed RVOT ablation cases, the findings of this study should be interpreted as exploratory and hypothesis‐generating. In addition, the mapping results may be influenced by mapping system settings and catheter position, which could affect the temporal and spatial relationship between FD and NF annotations.

## Funding

The authors have nothing to report.

## Disclosure

Registry and Registration no.: 1457.

## Ethics Statement

The study was approved by the hospital's institutional review board.

## Consent

All patients provided written informed consent for the ablation procedure and enrollment in the ablation registry.

## Conflicts of Interest

The authors declare no conflicts of interest.

## Data Availability

The data that support the findings of this study are available on request from the corresponding author. The data are not publicly available due to privacy or ethical restrictions.
